# Engineering Phosphatidylserine Containing Asymmetric Giant Unilamellar Vesicles

**DOI:** 10.3390/membranes14090181

**Published:** 2024-08-23

**Authors:** Jake McDonough, Trevor A. Paratore, Hannah M. Ketelhohn, Bella C. DeCilio, Alonzo H. Ross, Arne Gericke

**Affiliations:** Department of Chemistry and Biochemistry, Worcester Polytechnic Institute, 100 Institute Rd., Worcester, MA 01609, USAtaparatore@wpi.edu (T.A.P.); hmketelhohn@wpi.edu (H.M.K.); bcdecilio@wpi.edu (B.C.D.); aross@wpi.edu (A.H.R.)

**Keywords:** phosphatidylserine, asymmetric lipid bilayer, asymmetric giant unilamellar vesicle (GUV), membrane asymmetry

## Abstract

The plasma membrane lipid distribution is asymmetric, with several anionic lipid species located in its inner leaflet. Among these, phosphatidylserine (PS) plays a crucial role in various important physiological functions. Over the last decade several methods have been developed that allow for the fabrication of large or giant unilamellar vesicles (GUVs) with an asymmetric lipid composition. Investigating the physicochemical properties of PS in such asymmetric lipid bilayers and studying its interactions with proteins necessitates the reliable fabrication of asymmetric GUVs (aGUVs) with a high degree of asymmetry that exhibit PS in the outer leaflet so that the interaction with peptides and proteins can be studied. Despite progress, achieving aGUVs with well-defined PS asymmetry remains challenging. Recently, a Ca^2+^-initiated hemifusion method has been introduced, utilizing the fusion of symmetric GUVs (sGUVs) with a supported lipid bilayer (SLB) for the fabrication of aGUVs. We extend this approach to create aGUVs with PS in the outer bilayer leaflet. Comparing the degree of asymmetry between aGUVs obtained via Ca^2+^ or Mg^2+^ initiated hemifusion of a phosphatidylcholine (PC) sGUVwith a PC/PS-supported lipid bilayer, we observe for both bivalent cations a significant number of aGUVs with near-complete asymmetry. The degree of asymmetry distribution is narrower for physiological salt conditions than at lower ionic strengths. While Ca^2+^ clusters PS in the SLB, macroscopic domain formation is absent in the presence of Mg^2+^. However, the clustering of PS upon the addition of Ca^2+^ is apparently too slow to have a negative effect on the quality of the obtained aGUVs. We introduce a data filtering method to select aGUVs that are best suited for further investigation.

## 1. Introduction

Biological membranes are laterally heterogeneous (formation of domains with distinct lipid and protein compositions and hence unique physicochemical properties) and vertically asymmetric (different global and local compositions in the two bilayer leaflets). A longstanding question is to what extent domains present in one bilayer leaflet affect the physical properties of the membrane patch opposing that domain. In the plasma membrane, phosphatidylserine (PS) lipids are found exclusively in the inner leaflet of the lipid bilayer and its asymmetric distribution is crucial for many physiological functions [1,2]. Flippases maintain PS asymmetry by transporting the lipid from the outside to the inside of the cell [3], while scramblases diminish the asymmetric PS distribution [4,5]. It has been proposed that transient loss of membrane asymmetry might have significant biological functions [6,7]. In terms of lateral heterogeneities (domain formation), phosphatidylserine has been shown to form nanodomains [8] and is of major importance for the assembly and dynamics of caveolae [9]. The London group recently showed that loss of membrane asymmetry might promote formation of detergent resistant membranes [10]. 

Considering the many physiological functions of PS, it is important to understand the physiochemical underpinnings of PS function in asymmetric lipid bilayer systems. Giant unilamellar vesicles (GUVs) have been utilized for decades to investigate lipid properties and domain formation [11] however, their use was limited to symmetric GUVs (sGUVs) where the two bilayer leaflets have the same lipid composition. This changed with the introduction of the β-cyclodextrin lipid exchange method by the London group [12], which enabled the generation of asymmetric large unilamellar vesicles. In the following, a range of methods were introduced to fabricate large and giant unilamellar vesicles that exhibit different lipid compositions in the two bilayer leaflets [13,14,15,16]. Membrane mimics with an asymmetric distribution of PS have been obtained via enzymatic conversion [17], resorting of lipids between leaflets [18], droplet transfer method [19], inkjet printing [20] and lipid exchange with mβCD [12,21] (for a recent review of asymmetric lipid vesicle fabrication methods see Krompers and Heerklotz [14]). However, several of these techniques furnished membrane mimics with an incomplete membrane asymmetry and potential follow up experiments are difficult to design. 

The hemifusion method, developed by Enoki and Feigenson [22], is generally a very elegant approach to obtain asymmetric GUVs. The method involves the exchange of the outer leaflet of sGUVs via calcium cation induced fusion with a supported lipid bilayer (SLB). The inner leaflet of the resulting aGUV exhibits the lipid composition of the sGUV used for the fusion, while the outer leaflet has the lipid composition of the SLB. This method has been successfully used to study lipid phase diagrams, domain formation and driving forces for lipid order [23,24,25,26]. At present, this technique has not been applied to lipid systems with anionic lipids. 

The goal of this paper is to establish the hemifusion method for the fabrication of aGUVs with an anionic lipid composition in one leaflet of the lipid bilayer. Our experimental design results in aGUVs that exhibit PS in the outer leaflet so that interactions of peptides or proteins with the anionic lipid can be studied. In addition to adopting the Ca^2+^ based hemifusion method developed by Enoki and Feigenson [22], we expand the scope of the method to Mg^2+^ as the hemifusion initiating cation. The reason is that Ca^2+^ is known to cluster anionic lipids, including PS, and the use of Mg^2+^ might avoid complications due to domain formation in the supported lipid bilayer that is being fused with the sGUV. We obtain aGUVs with a high degree of lipid asymmetry and propose a data filtering method that allows for the selection of aGUVs with complete exchange. 

## 2. Materials and Methods

### 2.1. Materials

1,2-dioleoyl-sn-glycero-3-phosphocholine (DOPC), 1-palmitoyl-2-oleoyl-sn-glycero-3-phosphocholine (POPC), sodium 1-palmitoyl-2-oleoyl-sn-glycero-3-phospho-L-serine (POPS), and ammonium 1-palmitoyl-2-(dipyrrometheneboron difluoride)undecanoyl-sn-glycero-3-phospho-L-serine (TF-PS) were purchased from Avanti Polar Lipids (Alabaster, AL, USA), while 1,1-dioctadecyl-3,3,3,3-tetramethylindodicarbocyanine 4-chlorobenzenesulfonate (DiD) was purchased from Invitrogen (Waltham, MA, USA). Each reagent was obtained in powder form and used as received. A DiD stock solution was prepared by dissolving DiD powder in ethanol, while lipid stock solutions were dissolved in a solution of 2:1 chloroform:methanol (by volume). All stock solution was stored at −20 °C. The concentration of the fluorophore (DiD and TF-PS) stock solutions was determined using the Beer–Lambert relationship, approximated from absorbance measurements in methanol at 646 nm and 496 nm, respectively, and the corresponding extinction coefficients (ε). The ε of DiD was obtained from the reagent lot’s certificate of analysis (ε = 250,000 M^−1^cm^−1^), while the ε of TF-PS was obtained from Avanti Polar Lipids (ε = 97,000 M^−1^cm^−1^).

Nunc Lab-Tech II 4-well chambered coverglasses were purchased from Thermo Fisher (Waltham, MA, USA). Commercial artist-grade tracing paper was purchased from Amazon (Seattle, WA, USA). Liquinox was obtained from Alconox (White Planes, NY, USA). Sodium chloride, potassium chloride, calcium chloride, magnesium chloride, disodium ethylenediamine tetraacetic acid (Na_2_EDTA), 4-(2-hydroxyethyl)-1-piperazineethanesulfonic acid (HEPES), sucrose, and glucose were obtained in high purity grades either through Fisher Scientific (Fairlawn, NJ, USA) or Sigma Aldrich (St. Louis, MO, USA). HPLC grade chloroform, ethanol, isopropanol, and methanol were also obtained through Fisher Scientific (Fairlawn, NJ, USA). Deionized water (18.2 MΩ-cm) was obtained using a RODI (Aztec, NM, USA) high-purity water system. 

### 2.2. sGUV Preparation

sGUVs were prepared using the PAPYRUS method with tracing paper as described by Pazzi et al. [27]. In brief, a rectangular piece of tracing paper was first cleaned by submerging it in chloroform for 30 min, gently stirring the mixture every 10 min. This process was then repeated with a fresh volume of chloroform, after which it was repeated again with DI water. Next, the paper was dried in a vacuum oven. Once dry, a 6.35 mm diameter circular cutout was removed from the paper and coated with a 10 μL solution containing 0.5 to 1 mM lipids dissolved in 2:1 chloroform:methanol (by volume) at the same mole ratio as the desired sGUVs. After applying this mixture, the cutout was dried in a vacuum oven. Next, it was placed in the bottom of a microwell plate. If the sGUVs were to be prepared in low-salt buffer, 150 μL 200 mM sucrose, 5 mM HEPES, pH 7.4 buffer was added to the well, causing vesicles to bud from the dried lipid film on the paper and into solution. The resulting sGUVs were collected 2 h later. For sGUVs being prepared in normal (physiological) ionic strength buffer, 142.5 μL 105 mM sucrose was first added to the well containing the paper cutout, followed by 7.5 μL 100 mM sucrose, 100 mM NaCl, 50 mM KCl, 25 mM HEPES, pH 7.4 buffer 10 min later. The sGUVs were then collected after 110 min.

To collect sGUVs after their formation, a pipette with a wide orifice tip was used to mix the solution in the well by aspirating and dispensing 100 μL of it 6 times. The solution was then removed. If the sGUVs were prepared in the higher salt buffer, the osmolality of their collected solution was then measured with an Advanced Instruments model 3300 Micro Osmometer (Norwood, MA, USA) Any buffers later added to these sGUVs were adjusted to within ±2 mOsm/kg this reading using the same protocol as described for the low-salt buffers. 

### 2.3. Coating of Cover Glasses

sGUV controls were imaged in chamber well plates with coverglass bottoms, which were coated with bovine serum albumin (BSA) to prevent the vesicles from sticking to the glass. To perform this coating on a chambered coverglass, it was first cleaned by rinsing its chambers with DI water three times, followed by ethanol two times, isopropanol once, then ethanol once. This rinsing was then repeated, ending with a final 3 rinses of DI water. Next, the coverglass was sonicated for 15 min in dilute Liquinox detergent (around 2% (*w*/*v*) in DI water) heated to 69 °C in a Branson 1510 water bath sonicator (Brookfield, CT). Once done, the coverglass was rinsed with DI water 12 times, followed by ethanol two times, isopropanol once, then ethanol once. This rinsing was then repeated with a final 12 rinses of DI water. The coverglass was dried under N_2_ gas and primed for BSA coating by filling each chamber with 0.1 M NaCl. After 40 min, 0.1 M NaCl was replaced with 1 mg/mL BSA in 0.1 M NaCl at pH = 5.0 to apply the BSA coating. After 3 or more hours, the coverglass was rinsed with DI water 12 times to remove excess BSA not bound to its surface. 

### 2.4. Solid Supported Lipid Bilayer (SLB) Preparation

SLBs were fabricated in chambered coverglasses using the vesicle fusion method first described by Brian and McConnell [28]. In this procedure, SUVs were first prepared by creating a 1 mM lipid mixture in 2:1 chloroform:methanol (by volume) with the lipids at the same mole ratios as the desired SLB. This solution was then dried down under N_2_ and resuspended in a 500 mM NaCl, 20 mM citrate, pH = 4.0 buffer to form multilamellar vesicles. Next, the multilamellar vesicles were subjected to six freeze/thaw cycles to obtain SUVs. Then, the suspension was sonicated using a Sonics Vibra-Cell VCX130 Ultrasonic Processor tip sonicator (Newton, CT, USA), where an amplitude of 50% was applied to the vesicles in 15 s pulses (15 s on, 15 s off) for 30 min. The resulting SUVs were then diluted fivefold in the buffer described above.

Before using the SUVs to form the SLBs, an unused chambered coverglass was first cleaned using the same method as described for the BSA-coated coverglasses, but in this case, after drying the cleaned coverglass with N_2_, it was plasma-cleaned for 1 m 20 s using a Mercator Control Systems LF-5 Plasma System with O_2_ gas. Immediately after plasma cleaning, 1 mL of the diluted SUVs was added to each chamber to begin SLB formation. In this process, the SUVs in each chamber settle to the bottom and fuse to form a planar bilayer. The high ionic strength and acidity of the buffer helps to overcome electrostatic repulsions that would inhibit fusing.

After 40 or more minutes, the unfused vesicle remnants were washed out by submerging the coverglass in 2.5 L of DI water and using a syringe to gently rinse each chamber two times with 10 mL DI water. Next, the coverglass was removed from the water, and liquid was gently removed from the top of each chamber to return their volumes to 1 mL. Finally, the water in each chamber was exchanged for either a 65 mM NaCl, 35 mM KCl, 25 mM HEPES, pH 7.4 buffer or a 100 mM NaCl, 100 mM KCl, 25 mM HEPES, pH 7.4 buffer depending on if the SLB was being prepared in low or high-salt buffer respectively. To do so, 1 mL of the buffer was added to a chamber dropwise, then 1 mL was gently removed from a location in the chamber different than where the buffer was added. This process was repeated for each chamber 16–24 times, stopping once the chamber’s osmolality was equal to the buffer used to prepare the sGUVs within ±2 mOsm/kg, measured using The Advanced Micro Osmometer, Model 3300. SLBs were used immediately after being prepared. 

### 2.5. Monitoring Domain Formation in SLBs

SLBs were prepared in low or higher salt buffer with DOPC/POPS/TF-PS (69.9/30/0.1 mol%). These were then imaged before and after the introduction of 3 mM Ca^2+^ or Mg^2+^ to the aqueous phase by the addition of 400 μL 11 mM MCl_2_ (M = Ca^2+^ or Mg^2+^), 42 mM NaCl, 42 mM KCl, 25 mM HEPES, pH 7.4 buffer (“low-salt” buffer) or 400 μL 11 mM MCl_2_ (M = Ca^2+^ or Mg^2+^), 108 mM NaCl, 76 mM KCl, 25 mM HEPES, pH 7.4 buffer (“high-salt” buffer). 50 μL 200 mM sucrose, 5 mM HEPES, pH 7.4 buffer (if in low-salt buffer) or 100 mM sucrose, 100 mM NaCl, 50 mM KCl, 25 mM HEPES, pH 7.4 buffer (if in high-salt buffer) was added to the SLBs at the same time as the cations to mimic the conditions of hemifusion. For each SLB, images were taken every 20 min following the additions at the same locations on the SLB. 

### 2.6. Asymmetric GUV (aGUV) Preparation

Asymmetric GUVs were prepared using an adapted form of the hemifusion method described by Enoki et al. [22] In this procedure, 50 μL DOPC/DiD (99.9/0.1 mol%) sGUVs in low or high-salt buffer was added to a chamber containing a DOPC/POPS/TF-PS (69.9/30/0.1 mol%) SLB in low or high-salt buffer, respectively. After waiting 10 min for the sGUVs to settle to the bottom of the chamber, hemifusion between the sGUVs and SLB was induced by adding 5.5 mM Ca^2+^ or Mg^2+^ to the chamber via the addition of 400 μL 20 mM MCl_2_ (M = Ca^2+^ or Mg^2+^), 35 mM NaCl, 35 mM KCl, 25 mM HEPES, pH 7.4 (if in low-salt buffer) or buffer 20 mM MCl_2_ (M = Ca^2+^ or Mg^2+^), 100 mM NaCl, 70 mM KCl, 25 mM HEPES, pH 7.4 (if in high-salt buffer). After waiting 23 min for hemifusion and lipid exchange to occur between the membranes, fission of the GUVs from the SLB was induced by adding 6 mM EDTA to the chamber via the addition of 600 μL 20 mM Na_2_EDTA, 35 mM NaCl, 35 mM KCl, 25 mM HEPES, pH 7.4 buffer (for low-salt buffer) or 20 mM Na_2_EDTA, 100 mM NaCl, 76 mM KCl, 25 mM HEPES, pH 7.4 buffer (for high-salt buffer). After waiting 10 min for fission to complete, the resulting aGUVs were harvested. 

### 2.7. Fluorescence Imaging

All GUVs and SLBs were imaged using a Zeiss LSM 510 confocal microscope (Oberkochen, Germany). SLBs and aGUVs were imaged in the coverglass wellplate they were prepared in, while sGUVs were imaged in BSA-coated coverglasses by adding 50 μL of the sGUVs to the chamber, followed by 550 μL 200 mM glucose, 5 mM HEPES, pH 7.4 buffer or to 550 μL 100 mM glucose, 100 mM NaCl, 50 mM KCl, 25 mM HEPES, pH 7.4 buffer if they were prepared in low or high-salt buffer, respectively. Adding the glucose buffer after adding the GUVs helps the vesicles settle on the bottom and provides a better imaging contrast. When imaging vesicles, only those with no deformities (e.g., multilamellarity, tubules, non-spherical morphology) were imaged. All GUV images were obtained by using a plan-apochromat 63× oil immersion objective, while SLB images were taken either with this lens, or a plan-apochromat 10× air objective. Unless otherwise specified, all GUV images were taken by capturing a single frame at the equator of the vesicle, and later processed by applying a Gaussian blur of radius 1. Meanwhile, all 63× lens images of SLBs were taken as Z-stacks, with the Z-slices spanning from right above to right below the membrane. All Z-stacks in this study were converted into composite 2D images, where each pixel in the image has the average intensity of all the Z-slices at that pixel. Certain Z-stacks of SLB domains were taken at a low detector gain, and later, digitally brightened to avoid image artifacts. All 10× lens images of SLBs were single frames that were digitally brightened as well. 

### 2.8. Analysis of the Fluorescence Intensities

Using the analysis method developed by Enoki et al. [22], the fluorescence intensities of aGUVs were compared to those of sGUVs to evaluate the amount of lipid exchange each aGUV underwent during hemifusion. This analysis first involved deriving an intensity value for the DiD and TF-PS emissions of each aGUV. To do so, ImageJ macros generously provided by Thais Enoki and Gerald Feigenson (personal communication; used in [22]) were used to find the maximum DiD and TF-PS emission intensity along a ray going from the center of the vesicle outwards. This measurement was repeated for 359 more rays, with each angled in a different manner such to capture all areas of the vesicle. The maximum intensities captured by each ray were then averaged together to get an average maximum DiD intensity and average maximum TF-PS intensity for the vesicle. Going forward, these values will be referred to as “DiD intensity” and “TF intensity” respectively. In addition to being found for each aGUV, the DiD or TF intensity was also determined for sGUVs with a composition matching the theoretical inner or outer leaflet composition of the aGUVs.

Before performing any further analysis with each vesicle’s DiD or TF intensity, outliers were removed from the data set. An outlier was defined as a vesicle that had a DiD or TF intensity outside the range of their corresponding average DiD or TF intensity ± double the standard deviation. This average and standard deviation were taken from the vesicle’s sample, meaning all data for vesicles of that type, depending on composition (i.e., aGUV, sGUV with DiD, or sGUV with TF-PS) and buffer composition (i.e., low or high-salt buffer). An aGUV found to be an outlier for one intensity (DiD or TF) value but not the other was considered an outlier, and both intensities were completely discarded. As with this outlier removal, all future analyses were performed separately for vesicles in low and high-salt buffers, meaning intensities from vesicles in low-salt buffer were never compared to those from vesicles in high-salt buffer and vice versa.

A vesicle’s DiD or TF intensity essentially represents the average emission intensity of DiD or TF-PS across it, which is chiefly dependent on the fraction of DiD or TF-PS in the vesicle. aGUVs that experienced full outer leaflet exchange with the SLB should have half the fraction of DiD and TF-PS as their sGUV counterparts, meaning their DiD and TF intensities should be half that of the sGUVs. This reasoning was used to determine which aGUVs experienced complete outer leaflet exchange. In this analysis, an aGUV was considered to have undergone this complete exchange if both of the following were true:

1. Its DiD intensity fell within the range of the halved maximum and halved minimum DiD intensity of the corresponding sGUVs.
2. Its TF intensity fell within the range of the halved maximum and halved minimum TF intensity of the corresponding sGUVs.

When both of these conditions were met, it means the aGUV gave a DiD and TF intensity that was in line with what is expected from a population of vesicles with half the DiD and TF-PS content of the sGUVs. An aGUV that met these criteria is referred to as a “range-passing” aGUV, while those that did not are “range-failing”.

The DiD and TF intensities of the aGUVs were also used to find each vesicle’s outer leaflet DiD % exchange and TF % exchange. These values equal the aGUV’s DiD or TF intensity (I_a_) divided by the halved average DiD or TF intensity of the corresponding sGUVs (I_c_), expressed as a percentage:

$$\mathrm{Exchange}\;\mathrm{Percent}=\frac{I_{a}}{I_{c}/2}\times 100\%$$

An aGUV having close to a 100% outer leaflet DiD % exchange and TF % exchange indicates that its DiD and TF intensities closely matched the halved average intensities for the sGUVs. This situation suggests that the aGUV underwent a mostly complete outer leaflet exchange.

## 3. Results and Discussion

### 3.1. PS Domain Formation in the Presence of Ca^2+^ or Mg^2+^

**Figure 1:**
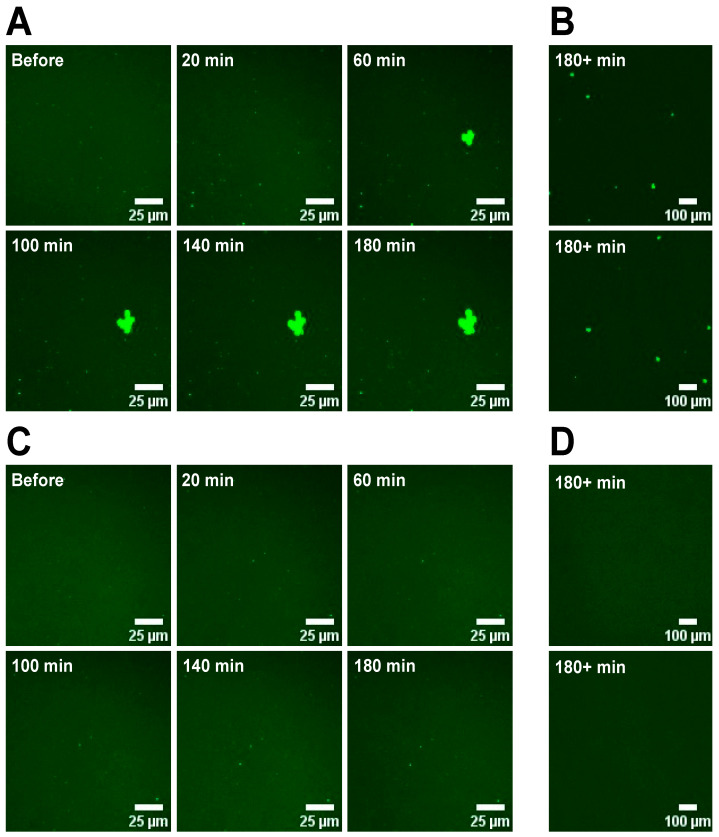
Time evolution of Ca^2+^- or Mg^2+^-induced domain formation in DOPC/POPS/TF-PS (69.9/30/0.1 mol%) SLBs. (**A**) 63× magnification SLB images at the same location just before and at 20 to 180 min after Ca^2+^ was introduced to the aqueous phase. A large domain rich in TF-PS appears as a bright green spot that increases in size over time. Images were taken at a low detector gain and are digitally brightened. (**B**) 10× magnification images of the SLB from panel (**A**) at two separate areas 180 or more minutes after adding Ca^2+^. With this lower magnification we can observe the formation of many such domains as presented in Panel (**A**). (**C**,**D**) Same as in panels (**A**,**B**), but with 3 mM Mg^2+^ present in the aqueous phase in place of Ca^2+^. After the addition of the bivalent cation, the buffer composition is 3 mM MCl_2_ (M= Ca^2+^ or Mg^2+^), 102 mM NaCl, 92 mM KCl, 3 mM sucrose, 25 mM HEPES, pH = 7.4.

In our previous work, we demonstrated that the addition of Ca^2+^ to PS containing supported lipid bilayers (SLBs) resulted in the formation of macroscopic domains due to PS/Ca^2+^ clustering [29]. For the hemifusion process, this poses the potential challenge that the sGUVs fuse with a macroscopically heterogeneous SLB. As a result, some aGUVs could exhibit excess PS (sGUVs fuse with a PS domain), while others have little or no PS due to fusion with SLB regions devoid of PS. To test the likelihood of this scenario occurring and to explore whether Mg^2+^ might be a useful alternative to Ca^2+^, a series of experiments were performed in which SLBs were imaged before and at various time points after the addition of Ca^2+^ or Mg^2+^. Figure 1 shows the time-dependent domain formation in POPS/DOPC (30:70 mol%) SLBs after the addition of Ca^2+^ or Mg^2+^, respectively. After the addition of Ca^2+^, macroscopic domain formation occurs within a time window of 20–60 min. The overview image (Figure 1B) shows multiple domains at the endpoint of the experiment across the field of view. For the fabrication of aGUVs using the hemifusion method, the fusion is stopped 10 min after the addition of Ca^2+^ by adding EDTA. This is well before the time point at which we observe Ca^2+^-induced PS domain formation, i.e., we expect that domain formation will not negatively impact the uniform formation of aGUVs. Upon the addition of Mg^2+^ to the POPS/DOPC SLB, we do not observe macroscopic domain formation for the entire duration of the experiment. We are exploring the usefulness of Mg^2+^ for hemifusion initiation because we have found for other anionic lipids that domain formation upon the addition of Ca^2+^ occurs significantly faster than what is found here for PS (Paratore and Gericke, unpublished results). Therefore, to investigate more complex lipid mixtures (PS + other anionic lipids) in aGUVs, we may have to switch to Mg^2+^.

**Figure A1:**
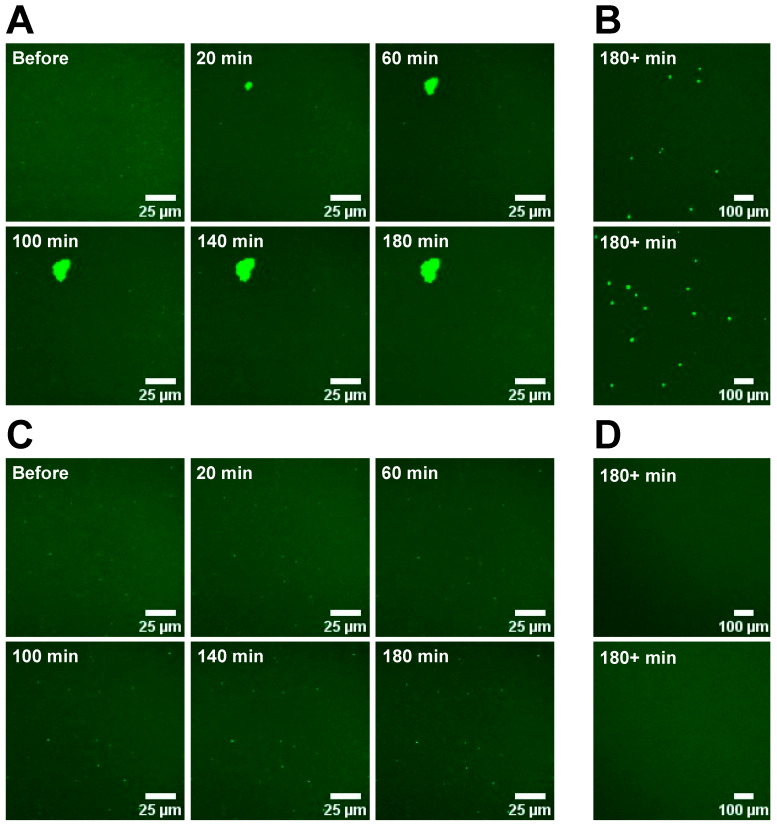
Time evolution of Ca^2+^ or Mg^2+^ induced domain formation in DOPC/POPS/TF-PS (69.9/30/0.1 mol%) SLBs at low salt concentration. (**A**) 63× magnification SLB images at the same location just before and at 20 to 180 min after Ca^2+^ was introduced to the aqueous phase. A large domain rich in TF-PS appears as a bright green spot that increases in size over time. Images were taken at a low detector gain and digitally brightened. (**B**) 10× magnification images of the SLB from panel A at two separate areas 180 or more minutes after adding Ca^2+^. With this lower magnification we can observe the formation of many such domains as presented in Panel (**A**). (**C**,**D**) Same as in panels (**A**,**B**), but with 3 mM Mg^2+^ present in the aqueous phase in place of Ca^2+^. After the addition of the bivalent cation, the buffer composition is 3 mM MCl_2_ (M = Ca^2+^ or Mg^2+^), 56 mM NaCl, 36 mM KCl, 7 mM sucrose, 24 mM HEPES, pH = 7.4.

In many studies, aGUVs are characterized for low-salt conditions, since the sGUVs required for the fabrication of aGUVs (independent of the method used) are easier to obtain via the electroformation method at low ionic strengths (though recently methods have been introduced that utilize the electroformation method at higher ionic strengths). While low ionic strength experiments for zwitterionic lipid mixtures are probably less problematic, we believe that experiments involving anionic lipids should be carried out at or near physiological conditions. Nevertheless, we repeated the experiments shown in Figure 1 at reduced salt concentrations (Appendix A Figure A1). We find the onset of macroscopic domain formation in the presence of Ca^2+^ to be slightly earlier (small domain formation at 20 min) than what we observed for the higher salt concentration. For Mg^2+^, we find also for this salt concentration no macroscopic domain formation for the duration of the experiment.

We also explored the effect of chain composition of the zwitterionic lipid on the observed PS domain formation in the presence of Ca^2+^ or Mg^2+^ and did not find significant differences between POPC and DOPC containing lipid mixtures (not shown). 

### 3.2. Fabrication and Characterization of aGUVs Obtained by Bivalent Cation Initiated Hemifusion

The fabrication of aGUVs via the hemifusion method involves the fusion of sGUVs with an SLB. The extent of the fusion is followed by monitoring the fluorescence intensity of labeled sGUVs vs. the corresponding intensity of the formed aGUVs. A successful fusion, where the outer leaflet of the sGUVs is completely exchanged with the lipids present in the SLB, should lead to a ~50% intensity in the aGUVs relative to the sGUVs. The aGUV has DOPC in the inner leaflet, while the outer leaflet contains the DOPC/POPS lipid mixture. While not part of this study, the “inside/out” approach allows us to add later species that interact with the PS component (e.g., proteins). For our experiments, the sGUVs used for the fusion exhibited a DOPC/DID (99.9/0.1 mol%) lipid mixture, while the SLB was composed of DOPC/POPS/TF-PS (69.9/30/0.1 mol%). 

**Figure 2:**
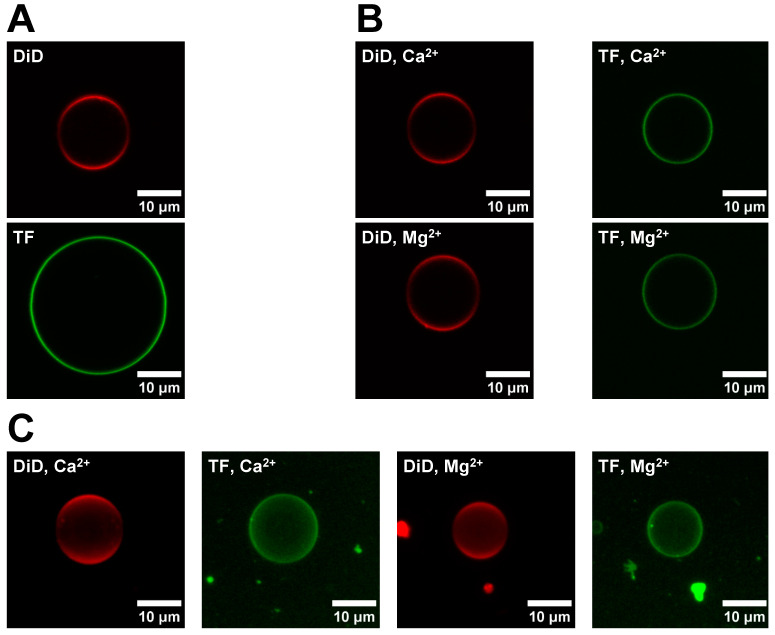
Comparison of sGUV and corresponding aGUV images. (**A**) Images of a DOPC/DiD (99.9/0.1 mol%) and DOPC/POPS/TF-PS (69.9/30/0.1 mol%) sGUV. sGUVs contain 100 mM sucrose, 100 mM NaCl, 50 mM KCl, 25 mM HEPES, pH = 7.4 and are imaged in a 100 mM glucose buffer of equal ionic strength, pH, and osmolality. (**B**) Images of aGUVs formed via hemifusion with Ca^2+^ or Mg^2+^. For each aGUV, the DiD (red) and TF (green) channels are shown separately. aGUVs are in 4 mM CaCl_2_ (or MgCl_2_), 6 mM Na_2_EDTA, 100 mM NaCl, 84 mM KCl, 2 mM sucrose, 25 mM HEPES, pH = 7.4. (**C**) Composite images of Z-stacks taken of a Ca^2+^- and Mg^2+^-formed aGUV in the same buffer as those in panel (**A**). Please note that the reduced intensity around the equator line in the DID (red) channel is due to polarization effects. Each fusion experiment was repeated four times. “DiD” sGUV: DOPC/DID 99.9/0.1 mol%; “TF” sGUV: DOPC/POPS/TF-PS 69.9/30/0.1 mol%.

**Figure A2:**
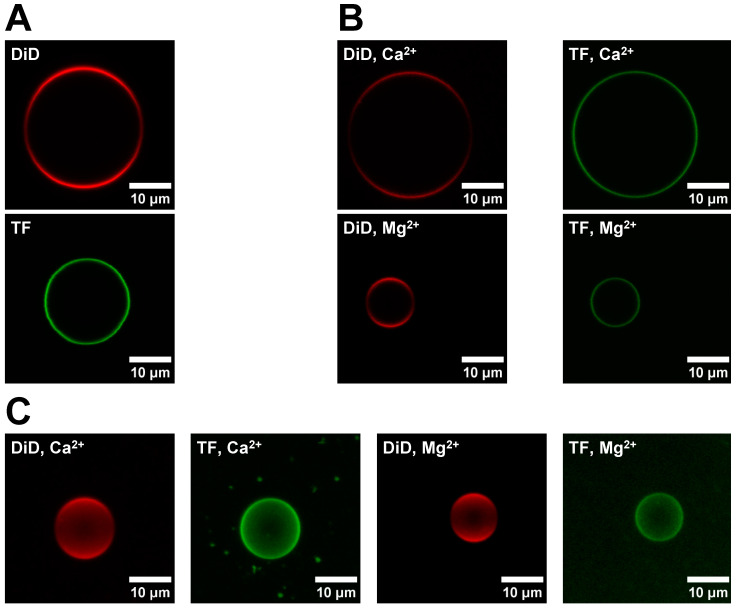
Images of sGUVs and aGUVs in low-salt buffer. (**A**) Images of a DOPC/DiD (99.9/0.1 mol%) and DOPC/POPS/TF-PS (69.9/30/0.1 mol%) All vesicles contain 200 mM sucrose, 5 mM HEPES, pH 7.4. sGUVs are imaged in 200 mM glucose, 5 mM HEPES, pH = 7.4. (**B**) Images of aGUVs formed via hemifusion with Ca^2+^ or Mg^2+^. For each aGUV, the DiD (red) and TF (green) channels are shown separately. aGUVs are measured in low-salt buffer with a final composition of 4 mM CaCl_2_ (or MgCl_2_), 6 mM Na_2_EDTA, 49 mM NaCl, 34 mM KCl, 5 mM sucrose, 25 mM HEPES, pH = 7.4. (**C**) Composite images of Z-stacks taken of a Ca^2+^- and Mg^2+^-formed aGUV in the same buffer as those in panel (**A**). Please note that the reduced intensity around the equator line in the DID (red) channel is due to polarization effects. This fusion experiment was performed eight times with Ca^2+^ and 18 times with Mg^2+^.

Figure 2A shows representative images for sGUVs formed with a DOPC/DID (red channel) and a DOPC/POPS/TF-PS (green channel) lipid mixture, respectively. The DOPC/POPS/TF-PS sGUVs are not used for the fusion experiment itself but are required to evaluate the completeness of the lipid exchange in the obtained aGUVs (see below for details). In Figure 2B the corresponding images are shown for aGUVs obtained via hemifusion with Ca^2+^ or Mg^2+^, respectively. As expected, the aGUVs show a reduced intensity for both channels relative to the sGUVs. Figure 2C shows composite images of the aGUVs. The corresponding images for sGUVs and aGUVs formed in a buffer with a low salt concentration are shown in Appendix B Figure A2. For both buffer conditions, approximately 5–20% of sGUVs underwent hemifusion to become aGUVs. None of the z-stacks had any major spots or holes, meaning the aGUVs are laterally homogenous with respect to each fluorophore. Note that all DID images displayed lower intensity around the GUV equator, which is due to a polarization artefact and not indicative of lateral asymmetries.

**Figure 3:**
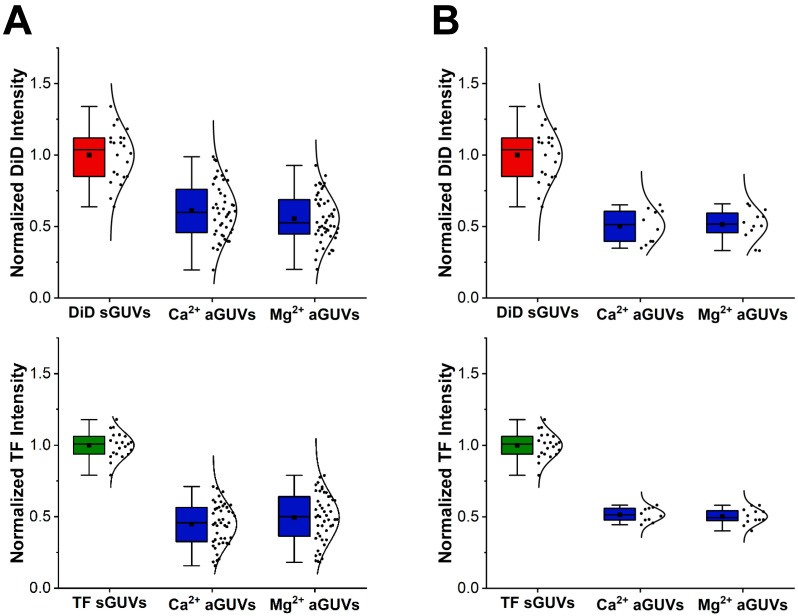
Intensity comparisons between sGUVs and aGUVs. (**A**) Intensities of Ca^2+^- and Mg^2+^-formed aGUVs compared to those of sGUVs with compositions equal to the theoretical inner leaflet composition (DiD sGUVs, graphed in red) or theoretical outer leaflet composition (TF sGUVs, graphed in green) of the aGUVs (all graphed in blue). Both symmetric and asymmetric GUVs contain 100 mM sucrose, 100 mM NaCl, 50 mM KCl, 25 mM HEPES, pH = 7.4. Unlike sGUVs, which are imaged in an isotonic glucose buffer of equal ionic strength and pH, aGUVs are imaged in a solution of 4 mM CaCl_2_ (or MgCl_2_), 6 mM Na_2_EDTA, 100 mM NaCl, 84 mM KCl, 2 mM sucrose, 25 mM HEPES, pH = 7.4. All intensities are normalized by the average of the corresponding sGUVs. Averages are shown as squares on each boxplot. (**B**) Same as panel (**A**), but after removing all range-failing aGUVs (spread of aGUV intensities is half of the spread observed for the sGUVs). “DiD” sGUV: DOPC/DID 99.9/0.1 mol%; “TF” sGUV: DOPC/POPS/TF-PS 69.9/30/0.1 mol%.

**Figure A3:**
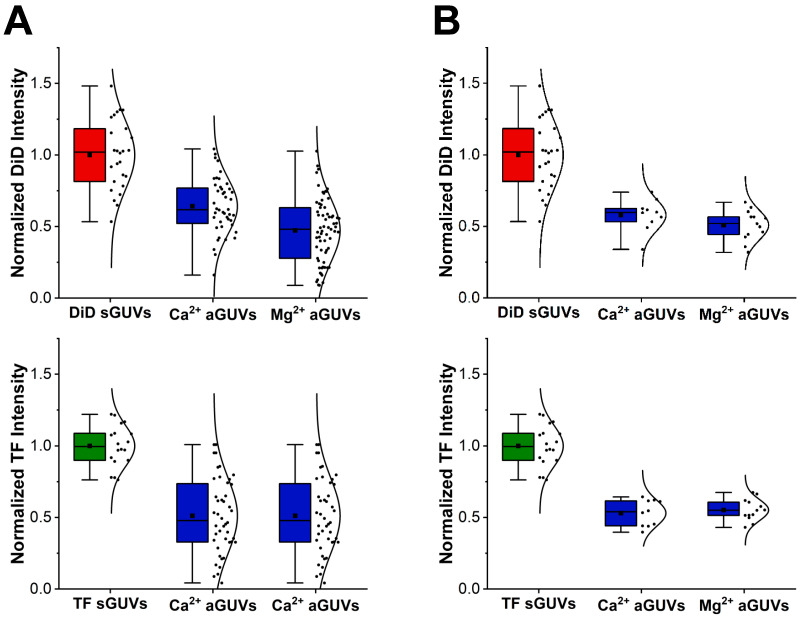
Intensity comparisons between sGUVs and aGUVs in low-salt buffer. (**A**) Intensities of Ca^2+^- and Mg^2+^-formed aGUVs compared to those of sGUVs with compositions equal to the theoretical inner leaflet composition (DiD sGUVs) or theoretical outer leaflet composition (TF sGUVs) of the aGUVs. All vesicles contain 200 mM sucrose, 5 mM HEPES, pH 7.4. sGUVs are imaged in 200 mM glucose, 5 mM HEPES, pH = 7.4. aGUVs are measured in low-salt buffer with a final composition of 4 mM CaCl_2_ (or MgCl_2_), 6 mM Na_2_EDTA, 49 mM NaCl, 34 mM KCl, 5 mM sucrose, 25 mM HEPES, pH = 7.4. All intensities are normalized by the average of the corresponding sGUVs. Averages are shown as squares on each boxplot. (**B**) Same as panel (**A**), but after removing all range-failing aGUVs (spread of aGUV intensities is half of the spread observed for the sGUVs).

To verify the composition of the prepared aGUVs, their DID and TF fluorescence intensities were compared to those of the corresponding sGUVs. This data is shown in Figure 3A for aGUVs obtained in physiological ionic strength buffer, while in the Appendix B Figure A3A the corresponding data are shown for a low ionic strength buffer. In all cases, the aGUVs were characterized by normalized DID and TF fluorescence intensities that were close to half of the corresponding sGUV fluorescence intensities, ranging from 0.45 to 0.64 (a value of 0.50 represents a complete exchange of the outer leaflet sGUV for the lipids in the SLB). This result demonstrates that that the aGUVs have on average the expected composition. However, all aGUV normalized fluorescence intensity distributions showed a considerable spread, each having a range of at least 0.55. This means that though the aGUVs give the expected composition on average, the exact composition of individual vesicles varies considerably. When comparing the spreads of different aGUV intensity distributions, we find that those from aGUVs in normal ionic strength buffer were tighter than those in low ionic strength buffer, with ranges that were 1.1 to 3.2 times smaller than the corresponding distributions in low ionic strength buffers. Additionally, DID intensity distributions were generally broader across both aGUVs and sGUVs, with ranges that were on average 1.5 times larger than the corresponding TF intensity distributions.

The DOPC/DID and DOPC/PS/TF-PS sGUVs show some intensity spread, which is probably due to variations in the fluorophore concentrations across the sGUV population. To account for these variations and to select for the aGUVs that have intensities within a range that can be expected based upon the intensity spread observed for the respective sGUV population, aGUV intensities were filtered to remove all range-failing vesicles. The acceptable range for the aGUV intensities is half the spread observed for the corresponding sGUVs (since for the aGUVs the intensity represents only one leaflet rather the two leaflets in the sGUVs). This filtering selected for aGUVs that had the expected composition with the same degree of compositional variability as the sGUVs. When applying this filtering, 24% in normal ionic strength (Figure 3B) and 21% of aGUVs in low-salt buffer (Appendix B Figure A3B) passed. We did not observe differences in the passing percentage for aGUVs formed via Ca^2+^ or Mg^2+^ initiated hemifusion. The average normalized intensities after filtering were even closer to being half of the sGUV averages, ranging from 0.50 to 0.58. Obviously, the new distributions were tighter than the corresponding unfiltered distributions. Overall, these results demonstrate that the filtered aGUVs are on average the expected composition, with very little variability between individual vesicles. 

**Figure 4:**
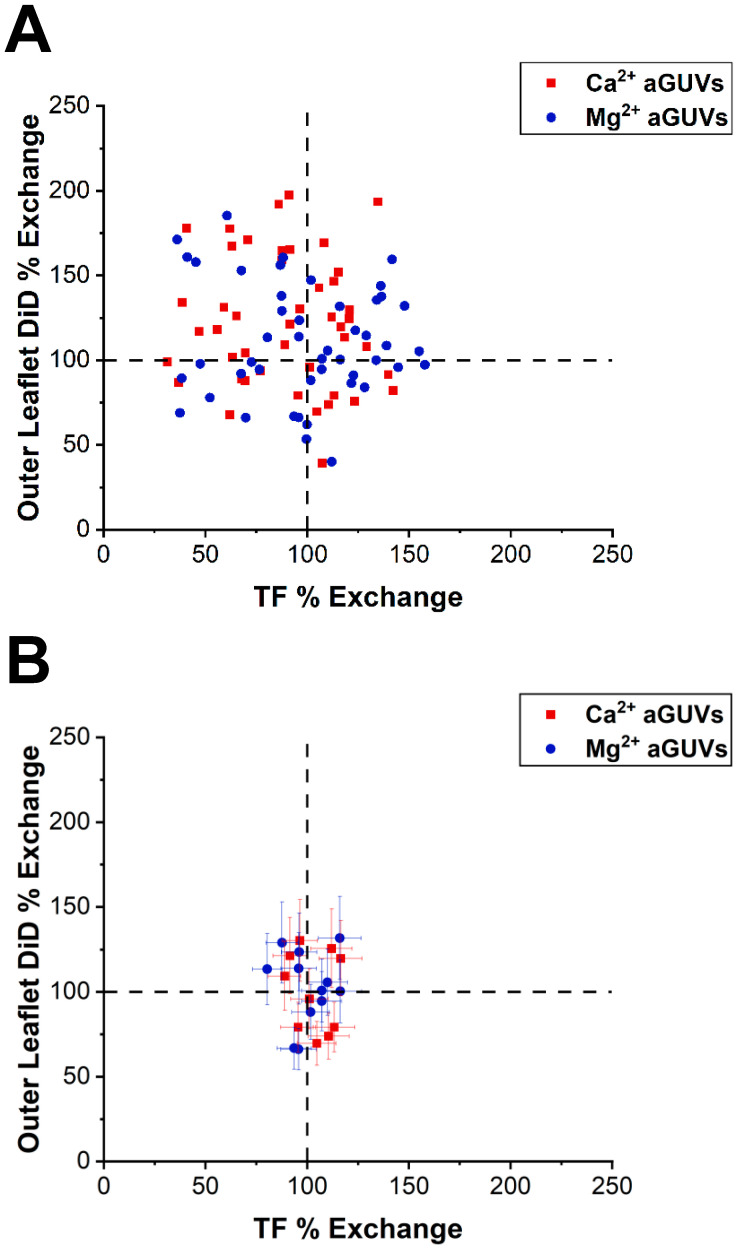
Correlation between DID and TF-PS outer leaflet exchange. (**A**) Outer leaflet DiD % exchange vs. TF % exchange for Ca^2+^- and Mg^2+^-formed aGUVs. aGUVs contain 100 mM sucrose, 100 mM NaCl, 50 mM KCl, 25 mM HEPES, pH = 7.4 and are imaged in 4 mM CaCl_2_ (or MgCl_2_), 6 mM Na_2_EDTA, 100 mM NaCl, 84 mM KCl, 2 mM sucrose, 25 mM HEPES, pH = 7.4. Error bars are omitted for clarity. (**B**) Same plot as in panel (**A**), but after removing all range-failing aGUVs. Error bars are calculated via a propagation of uncertainty using standard deviations.

**Figure A4:**
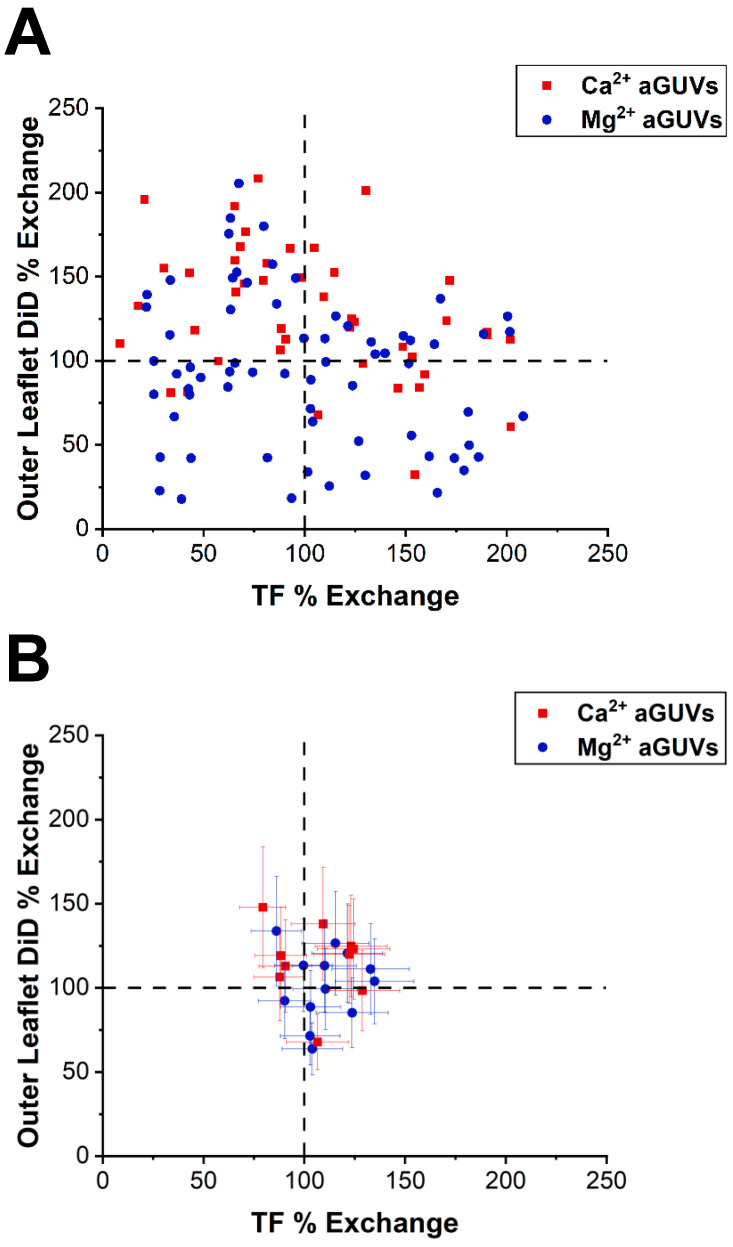
Correlation between DID and TF outer leaflet exchange in low-salt buffer. (**A**) Outer leaflet DiD % exchange vs. TF % exchange for Ca^2+^- and Mg^2+^-formed aGUVs. aGUVs are measured in low-salt buffer with a final composition of 4 mM CaCl_2_ (or MgCl_2_), 6 mM Na_2_EDTA, 49 mM NaCl, 34 mM KCl, 5 mM sucrose, 25 mM HEPES, pH = 7.4. Error bars are omitted for clarity. (**B**) Same plot as in panel (**A**), but after removing all range-failing aGUVs. Error bars are calculated via a propagation of uncertainty using standard deviations.

The intensities of each aGUV were compared to the average sGUV intensities to derive their outer leaflet DiD and TF% exchanges. Exchanges of 100% indicate that an aGUV has the expected intensity for that fluorophore given the theoretical composition. Therefore, an aGUV having 100% for both outer leaflet DiD and TF % exchange indicates that it is the expected composition. The exchanges for each aGUV are plotted in Figure 4 for physiological ionic strengths and in Figure A4 (Appendix B) for low ionic strength conditions. For both the unfiltered and filtered aGUV data, data points were scattered across the plots but were centered at around (100, 100). There was no clear correlation between outer leaflet DiD and TF % exchange, though some trends were observed. In both low- and normal-salt buffer, 2–3 times more Ca^2+^-formed aGUVs had outer leaflet DiD % exchanges above 100% than below. This relationship indicates that the majority of these aGUVs have larger than expected DiD intensities (“expected” being determined by the aGUVs’ theoretical compositions). We observe a tighter distribution around the 100/100 data point for the physiological ionic strength condition than for the low ionic strength.

**Figure 5:**
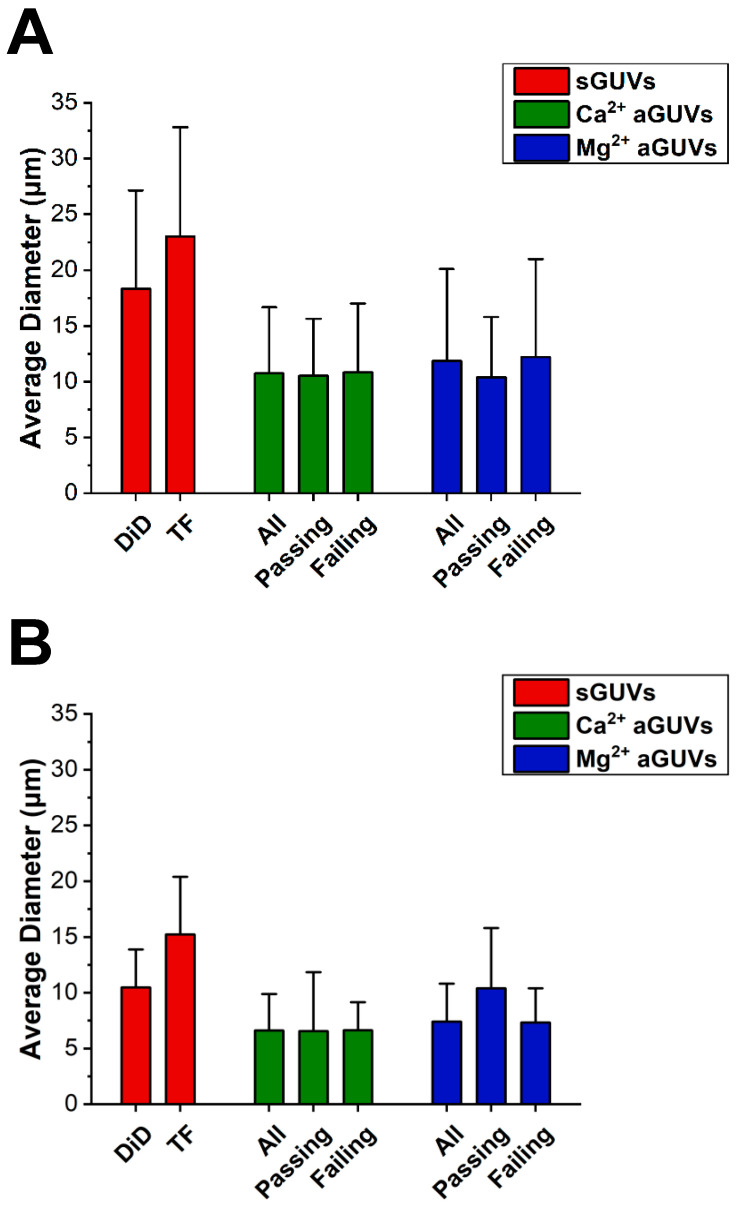
Diameter comparisons between sGUVs and aGUVs. (**A**) Average diameter of all Ca^2+^- and Mg^2+^-formed aGUVs, those that are range-passing, and those that are range-failing compared to that of sGUVs whose compositions match the theoretical inner leaflet composition (DiD sGUVs) or theoretical outer leaflet composition (TF sGUVs) of the aGUVs. Error bars show standard deviations. All vesicles contain 200 mM sucrose, 5 mM HEPES, pH 7.4. sGUVs are imaged in 200 mM glucose, 5 mM HEPES, pH = 7.4. aGUVs are measured in low-salt buffer with a final composition of 4 mM CaCl_2_ (or MgCl_2_), 6 mM Na_2_EDTA, 49 mM NaCl, 34 mM KCl, 5 mM sucrose, 25 mM HEPES, pH = 7.4. (**B**) Same as panel (**A**), but for vesicles in physiological ionic strength salt buffer. All vesicles contain 100 mM sucrose, 100 mM NaCl, 50 mM KCl, 25 mM HEPES, pH = 7.4. sGUVs are imaged in an isotonic glucose buffer of equal ionic strength and pH. aGUVs are imaged in 4 mM CaCl_2_ (or MgCl_2_), 6 mM Na_2_EDTA, 100 mM NaCl, 84 mM KCl, 2 mM sucrose, 25 mM HEPES, pH = 7.4. “DiD” sGUV: DOPC/DID 99.9/0.1 mol%; “TF” sGUV: DOPC/POPS/TF-PS 69.9/30/0.1 mol%.

To determine if vesicle size was influencing fluorescence data, the average diameter of the sGUVs and aGUVs was found, shown in Figure 5. On average, vesicles prepared in low ionic strength buffer had diameters 1.6 times larger than those at higher ionic strength. Additionally, in both low and normal ionic strength buffer, the sGUVs had diameters that were on average 1.8 times larger than the aGUVs. However, in both buffers, the average sGUV and aGUV diameters were within one standard deviation of each other. Similarly, the average diameters for aGUVs that were range-passing and range-failing were within one standard deviation of each other, indicating that diameter is not correlated with aGUVs being of the expected composition. It is interesting to note that the size of the sGUVs are for both lipid compositions (DID/DOPC and DOPC/POPS/TF-PS) smaller for the high ionic strength condition, i.e., it does not matter whether the lipid composition is zwitterionic or a combination of zwitterionic and anionic lipids. For both ionic strength conditions, aGUVs exhibit smaller sizes than the two types of sGUVs. The anionic sGUVs (DOPC/POPS/TF-POPS) are for both ionic conditions larger than the zwitterionic sGUVs (DOPC/DID). It can therefore be ruled out that the reduced size of the aGUVs relative to the zwitterionic sGUVs they were obtained from is due to the negative charge of the aGUVs. This relative shrinkage is likely inherent to the process of fabricating aGUVs via the hemifusion process.

## 4. Conclusions

We found the hemifusion method to be a suitable method to obtain high quality aGUVs with PS in the outer leaflet. Even though Ca^2+^ clusters PS, we did not find this to be a problem for the aGUV formation. The kinetics of macroscopic domain formation upon the addition of Ca^2+^ is apparently too slow to affect the aGUV formation. Since Mg^2+^ does not promote macroscopic domain formation, it might be a good alternative to induce hemifusion in instances where lipid mixtures are used that form macroscopic domains with Ca^2+^ faster than what we observed for PS. We did not find significant quality differences (in terms of degree of asymmetry and number of high-quality aGUVs obtained) between Ca^2+^ and Mg^2+^ induced hemifusion. The hemifusion method works equally well under low ionic strength and physiological ionic strength buffer conditions. While the average intensities in the respective leaflets of the aGUVs are near 50% of the corresponding sGUVs, we observe a fairly broad intensity distribution around this average value. Since the sGUV population that is being used for the hemifusion exhibits a range of intensities (presumably due to differences in the amount of fluorophore present in the vesicle), aGUVs with a completely exchanged outer leaflet should exhibit an intensity distribution that has a range half of what is observed for the sGUVs. Using a filtering method that accounts for this, we obtain a very narrow intensity distribution for both leaflets of the aGUV. In future studies utilizing this method, we will be able to select those aGUVs that show a leaflet intensity reflective of a 100% exchange of the outer leaflet. Experimental parameters like hemifusion time and temperature are likely to influence the degree of success of the hemifusion process. Whether or not the experimental parameters utilized for the lipid mixture investigated in this study carries over to other lipid mixtures with anionic lipids will need further exploration. Overall, we find the adapted hemifusion method to be a good choice for the fabrication of aGUVs with PS present in the outer leaflet.

## Data Availability

All data utilized in this study are stored on secure university servers and are made available upon request.

## Abbreviations

sGUV	Symmetric Giant Unilammelar Vesicle
aGUV	Asymmetric Giant Unilammelar Vesicle
DOPC	Dioleoylphosphahtidylcholine
POPS	Palmitoyloleoylphosphatidylserine
TF	TopFluor
DID	1,1-dioctadecyl-3,3,3,3-tetramethylindodicarbocyanine 4-chlorobenzenesulfonate
